# A UK study of the experiences, information needs and attitudes to clinical research among patients living with secondary breast cancer in the UK: A prospective co-developed study

**DOI:** 10.1016/j.breast.2025.104644

**Published:** 2025-11-12

**Authors:** Lesley Stephen, Janet Dunn, Claire Balmer, Nada Elbeltagi, Sophie Gasson, Ellen Copson, Carlo Palmieri

**Affiliations:** a Make 2nds Count Charity, Edinburgh, UK; b Warwick Clinical Trials Unit, University of Warwick, Coventry, UK; c Cancer Sciences Academic Unit, Faculty of Medicine, University of Southampton, Southampton, UK; d Institute of Systems, Molecular and Integrative Biology, Molecular and Clinical Cancer Medicine, University of Liverpool, Liverpool, UK; e The Clatterbridge Cancer Centre NHS Foundation Trust, Liverpool, UK

**Keywords:** Breast cancer, Metastatic, Clinical research, Attitudes, Experience

## Abstract

**Background:**

Clinical research is key to improving the outcomes of patients with metastatic breast cancer (MBC). However, participation is low, with little data on patients’ attitudes and experiences of clinical research. This study aimed to explore the experience and attitude of patients in accessing and participating in clinical research in the UK.

**Methods:**

An online survey, available between May and November 2021, was open to people living with MBC in the UK; this was complemented with by qualitative interviews.

**Findings:**

768 responses were received (766 female, 2 male); median age was 51–60 years with 235 (31 %) having de novo disease. 660 (86 %) respondents were confident in their understanding of clinical research. Discussion of participation in research with an oncologist was reported by 173 (23 %) respondents. Accessing new treatments was the most common reason for study participants wanting to take part in research, 737 (96 %). Of the 107 (14 %) respondents who had taken part in clinical trials, 77 (72 %) reported a positive experience. 276 (36 %) would consider travelling to participate in research and 430 (56 %) would be more likely to travel if expenses were met. Themes emerging from the qualitative interviews include ‘lack of information’, ‘barriers to participation’ and ‘participants research priorities’.

**Interpretation:**

This is the largest UK prospective study in regards to the views of MBC patients towards research. It demonstrates keenness to be involved in research, but participants face barriers as well as a lack of opportunity for participation. Key messages include importance of clinical staff in providing research information, need to develop patient accessible information, and to support travel costs. Improvements within the UK health care system are necessary to enable MBC patients to have equitable access to clinical research.

## Highlights

• A UK wide survey of metastatic breast cancer patients in relation to clinical research was performed.
• Only a small minority reported participation in a clinical trial.
• Preferred sources of information about trials: clinical staff and a patient friendly database.
• Most common reason for wanting to participate in a trial was accessing a new treatment.
• Most common given for not participating possible side effects and being unsure of the potential benefits.

## 1 Background

Globally, breast cancer is the second most common cancer with over two million new cases diagnosed and 684,996 deaths in 2020 [1]. The advances in the therapeutic management of metastatic breast cancer (MBC) have been built on robust, well designed clinical trials, and these latterly have led to the introduction of poly adenosine diphosphate-ribose polymerase inhibitors [2,3], anti-body drug conjugates to HER2 and Trop2 [4,5], cyclin dependant kinase 4/6 inhibitors [6], and immunotherapy [7], which in the context of clinical trials have resulted in improved outcomes. Evidence of increasing prevalence supports that women and men are living longer with their disease [8].

Access to well-designed clinical trials is a key recommendation within the 6th and 7th International consensus guidelines for the management of advanced breast cancer [9], and the ESMO clinical practice guideline for metastatic breast cancer encourages participation in clinical trials and that preference be given to enrolment onto a clinical trial, if available [10]. Healthcare systems such as the National Health Service (NHS) recognise the importance of research and the need to support it in the context of cancer care [9,11], as well as the need to empower patients to directly and proactively explore research opportunities [12].

Despite the recognised importance of offering and enabling access to clinical trials to cancer patients, they face real barriers to participation. These include the country in which the patient lives [13], eligibility criteria that can exclude potential participants [[14], [15], [16], [17]], and socio-economic factors which can influence the *likelihood* of participation such as income, ethnicity and the location, (urban vs rural), where a patient lives [10,11,18,19]. The accessibility of patient facing written materials in terms of readability, languages available and format such as for the visually impaired can also be a barrier [[12], [13], [14],[20], [21], [22]]. The absence of conversations about research between clinicians and patients, and a lack of systematic prioritisation of research within healthcare systems can further hamper patients being offered clinical trials [23]. Patient advocates have highlighted personal difficulties in both identifying and accessing clinical trials relevant to their specific cancer type and stage (personal communication). Given this lived experience and lack of formal data regarding the views and experience of those living with MBC in relation to clinical research, a study was co-developed between academics and a patient advocate living with MBC. The primary aim was to investigate the knowledge and experiences of people living with MBC in the UK regarding clinical research. Further objectives were to identify barriers to involvement in clinical research as well as ascertaining any information needs regarding involvement in research.

## 2 Methods

### 2.1 Study design

This was a co-developed and co-delivered study between a patient living with MBC and members of the National Cancer Research Institute (NCRI) Breast clinical study group. It was a mixed methods study consisting of an online survey and qualitative interviews carried out from a selection of survey respondents who consented to be interviewed.

### 2.2 Eligibility criteria

The inclusion criteria were male or female patients aged 18 or older with locally advanced or metastatic breast cancer based in the UK. The only exclusion criteria was the inability to complete the questionnaire, even with help from family member, carer or friend.

### 2.3 Sample size

At the time of the study design it was estimated 36,000 patients were living with MBC in the UK. The study aimed to recruit 10 % or more. The sampling technique was by self-selection.

### 2.4 Recruitment

Participants were recruited by self-identifying via posters and leaflets displayed at NHS sites, cancer support sites such as Maggie's Centres as well as local support groups, via charity websites of Make Seconds Count and Breast Cancer Now and social media. In addition, handouts with the survey details and links were also made available.

### 2.5 Consent

Participants who participated in answering the survey ticked to indicate consent and confirmed that they had been diagnosed with locally advanced or metastatic (secondary) breast cancer were included in the final analysis. Consent for the telephone interview was obtained when participants who had supplied contact details were first contacted by the qualitative researcher.

### 2.6 Delivery of survey

The survey was conducted online and administered on the Qualtrics platform between May and November 2021. A QR code was provided in online information and within all printed information, which gave direct access to the survey. At the start of the questionnaire a summary explaining the aims of the study and a further link to the patient information sheet were provided. Study participants who clicked on the link to the online survey were required to tick boxes to confirm that they understood the aims of the study, fulfilled the eligibility criteria, and that they provided informed consent. All eligible surveys were analysed irrespective of the number of questions completed.

### 2.7 Structure of survey

The survey contained closed and open questions which covered seven areas, these were: 1. Demographics, 2. Health status, 3. Experience of discussions about trials with health providers, 4. Experience of clinical trial participation, 5. Potential barriers to clinical trial participation, 6. Preferences for receiving information and 7.The effect of COVID-19 on cancer treatment. The full questionnaire is available in the supplementary methods.

### 2.8 Statistical analysis

Descriptive analysis of the closed questions responses and thematic analysis of data generated from the open-ended questions was applied.

### 2.9 Qualitative interviews

Survey respondents were invited to leave contact details if they were willing to participate in a follow-up interview. Phone or video interviewing was offered for ease of participation and due to previous COVID-19 related restrictions discouraging meeting people in person. Interviews by the same qualitative researcher (CB) using a topic guide (please refer to supplementary methods) took place between August and November 2021, and were transcribed verbatim. Data were extracted and stored in a separate database.

Thematic analysis was applied to the qualitative data. Each interview was scrutinised several times by an experienced researcher (CB). NVivo 12 was used to categorise and collate data and generate initial codes. Themes were then identified, reviewed and defined from the interviews. These were then examined and assessed by another researcher (SG) to ensure validity.

### 2.10 Ethical approval

The study was approved by Fulham Research Ethics Committee on the 27th April 2021 (REC Reference 21/LO/0232). Consent to anonymously reproduce interview quotes was given in writing by all interviewees. The study was conducted in accordance with the International Conference on Harmonization Good Clinical Practice guidelines and the provisions of the Declaration of Helsinki.

## 3 Results

Between 17th May and 30th November 2021, 834 individuals consented to take part in the online survey, with 768 respondents meeting the inclusion criteria.

### 3.1 Patient demographics

The characteristics of the eligible population are summarised in Table 1. 765 (99 %) were female with 708 (92 %) being of White UK ethnicity. The median age was 51–60 years which was also the largest represented age group (284 of 768; 37 %). 396 (51 %) of respondents were in some form of employment. 235 (31 %) had been diagnosed with de novo metastatic disease, 107 (14 %) had been living with metastatic breast cancer for ≥5years and 31 % diagnosed less than one year. The most common treatment received since diagnosis of metastatic disease was endocrine therapy with or without a targeted therapy (488 of 768; 64 %).

**Table 1:** Characteristics of survey respondents.

Characteristics	Frequency n (%)
**Gender**	
Male	2 (0.3)
Female	765 (99.6)
Not answered	1 (0.1)
**Age group**	
Under 30	10 (1.3)
31–40	104 (13.5)
41–50	225 (29.3)
51–60	284 (37.0)
61–70	114 (14.8)
71+	30 (3.9)
Not answered	1 (0.1)
**Ethnicity**	
White UK (English/Welsh/Scottish/Northern Irish/British)	708 (92.2)
Irish	9 (1.2)
Gypsy or Irish Traveller	1 (0.1)
White and Black Caribbean	5 (0.7)
White and Black African	1 (0.1)
White and Asian	3 (0.4)
Indian	6 (0.8)
Chinese	1 (0.1)
African	3 (0.4)
Any other Asian background	1 (0.1)
Any other mixed background	3 (0.4)
Any other white background	25 (3.3)
Any other ethnic group	1 (0.1)
Not answered	1 (0.1)
**Employment**	
Employed full-time	184 (24.0)
Employed part-time	161 (21.0)
Self-employed	51 (6.6)
Off sick	22 (2.9)
Unemployed	51 (6.6)
Unemployed due to health	18 (2.3)
Full-time housewife or husband	78 (10.2)
Retired	123 (16.0)
Retired due to ill health	69 (9.0)
Other (please specify)	6 (0.8)
Not answered	5 (0.7)
**Time since diagnosis**	
<1 year	235 (30.6)
1–2 years ago	160 (20.8)
2–3 years ago	121 (15.8)
3–4 years ago	93 (12.1)
4–5 years ago	51 (6.6)
>5 years ago	107 (13.9)
Not answered	1 (0.1)
**Treatments since being diagnosed with metastatic breast cancer**	
Surgery	224 (29.2)
Radiotherapy	339 (44.1)
Chemotherapy	459 (59.8)
Hormone therapy±targeted therapy	488 (63.5)
Anti-HER2 drugs	198 (25.8)
Immunotherapy	35 (4.7)
Other	133 (17.3)
Not answered	2 (0.3)

### 3.2 Knowledge and opportunities related to clinical trials

660 (86 %) reported knowing about clinical trials. The vast majority 591 (77 %) had not been invited to participate in a clinical trial, but 524 (68 %) of participants had themselves asked about trial participation (Table 2). Motivating factors reported for participation in a clinical trial were access to new treatments (737; 96 %), helping future patients (713; 93 %), playing more active role in own health (619; 81 %) and more frequent health check-ups (552; 72 %). The main reason given for not wanting to participate in a clinical trial were possible side effects of treatment (483; 63 %) and being unsure of the potential benefits (333; 43 %). Only 301 (39 %) of participants felt very involved in decision making about their treatment. Representative quotes from participants who took part in qualitative interviews regarding knowledge and opportunities of clinical trials are present in the supplementary data (Suppl results 1).

**Table 2:** Knowledge of trials and opportunities to participate in trials.

**Do you know what a clinical trial is?**	
No	19 (2.5)
Not sure	86 (11.2)
Yes	660 (85.9)
Not answered	3 (0.4)
**Has an oncologist ever raised taking part in a clinical trial?**	
Yes	173 (22.5)
No	591 (77.0)
Not answered	4 (0.5)
**Have you ever asked an oncologist to take part in a clinical trial?**	
Yes	243 (31.6)
No	524 (68.2)
Not answered	1 (0.1)
**What might encourage you to take part in a trial?**	
Early access to potential new treatment	737 (96.0)
Playing a more active role in own health	619 (80.6)
More frequent health check-ups	552 (71.9)
Helping future patients by taking part in research	713 (92.8)
**What things might stop you taking part in a clinical trial?**	
Cost	281 (36.6)
Travel	240 (31.3)
Being unsure of potential benefits	333 (43.4)
Not understanding what the trial is about	238 (31.0)
More visits to hospital	184 (24.0)
Possible side effects of treatment	483 (62.9)
Time off work	100 (13.0)
Other	60 (7.8)
**How involved do you feel in making decisions about your treatment?**	
Not at all involved	97 (12.6)
Slightly involved	359 (46.7)
Very involved	301 (39.2)
Not answered	11 (1.4)

#### 3.2.1 Information about clinical trials

When participants were asked if they were interested in finding out about clinical trials and how they would like to receive information 612 (80 %) reported wanting to receive information about clinical trials from their consultant and 467 (61 %) from a specialist nurse. 195 (25 %) of patients had searched a trials registry with 103 of 195 (53 %) not finding the information they required. Using a scale of 1 (hard) to 10 (easy), the mean ease of use of these trial registries as rated by those who had used them was 5.6. 612 (80 %) had not contacted any charity for advice on clinical trials. The vast majority of participants, 671 (87 %), indicated they would likely use a patient-friendly metastatic breast cancer trials registry, (Table 3).

**Table 3:** Information sources regarding clinical trials.

**If you were interested in finding out about clinical trials, how would you want to receive that information?**	
From a consultant	612 (79.7)
From a specialist nurse	467 (60.8)
From a friend/other patient	92 (12.0)
From a trials database	220 (28.7)
No preference	134 (17.5)
**Have you ever searched a trials registry? (Examples could be the National Cancer Institute registry or clinicaltrials.gov)**	
Yes	195 (25.4)
No	565 (73.6)
Not answered	8 (1.0)
**Did you find the information you were looking for?**	
Yes	74 (38.0)
No	103 (52.8)
Not answered	18 (9.2)
**How easy did you find it to use?**	
Mean (standard deviation)	5.6 (2.4)
Not answered	18 (9.2)
**How likely would you be to use a patient-friendly metastatic breast cancer trials registry?**	
Likely	671 (87.4)
Unlikely	71 (9.2)
Not answered	26 (3.4)
**Have you ever contacted any of the following organisations for advice on clinical trials?**	
Cancer Research UK (CRUK)	42 (5.5)
Breast Cancer Now (BCN)	50 (6.5)
None	612 (79.7)
Make 2nds count	41 (5.3)
Other	31 (4.0)

### 3.3 Experience of participating in a clinical trial and willingness to travel

Only 88 (11 %) of participants reported having undergone screening tests to see if they were eligible to take part in a clinical trial. With 107 (14 %) reporting that they had taken part in a clinical trial, of these individuals 77 (72 %) felt it was a positive experience. Only 33 (13 %) of those who participated in clinical trials had travel expenses reimbursed. 276 (36 %) of participants when asked if they would travel for a clinical trial, indicated a willingness to do so, this increased to 430 (56 %) if travel costs were covered. 306 (43 %) reported a willingness to travel worldwide for a clinical trial (Table 4).

**Table 4:** Experience of participating in a clinical trial and willingness to travel.

**Have you ever undergone screening tests (e.g. blood tests or scans) to see if you were eligible to take part in a clinical trial?**	
No	657 (85.6)
Yes	88 (11.5)
Not answered	23 (3.0)
**Have you taken part in a clinical trial?**	
No	641 (83.5)
Yes	107 (13.9)
Not answered	20 (2.6)
**Experience of taking part of the trial**	
Positive	77 (72.0)
Negative	7 (6.5)
Unsure	16 (15.0)
Not answered	7 (6.5)
**Were travel expenses reimbursed?**	
No	71 (66.4)
Yes	33 (30.8)
Not answered	3 (2.8)
**Approximately how much were you out of pocket because of taking part in a trial?**	
More than £500	5 (4.7)
£100-£500	12 (11.2)
Up to £100	7 (6.5)
I wasn't out of pocket	78 (72.9)
Not answered	5 (4.7)
**Have you ever tried to find out about clinical trials at centres other than your usual hospital or cancer centre?**	
Yes	120 (15.6)
No	619 (80.6)
Not answered	29 (3.8)
**Have you ever asked your oncologist to make enquiries for you at other cancer centres regarding trials for you?**	
Yes	56 (7.3)
No	678 (88.3)
Not answered	34 (4.4)
**Would you be willing to travel to another cancer centre specifically to take part in a clinical trial?**	
Yes	276 (35.9)
No	21 (2.7)
Maybe	444 (57.8)
Not answered	27 (3.5)
**How far would you be prepared to travel?**	
Up to 1 h	193 (26.8)
1–2 h	261 (36.3)
More than 2 h	257 (35.7)
Not answered	9 (1.3)
**Would you be prepared to travel abroad?**	
Yes, worldwide	306 (42.5)
Yes, to the USA	20 (2.8)
Yes, but only to a European country	84 (11.7)
No	301 (41.8)
Not answered	9 (1.3)
**Within the UK which modes of travel would you be likely to use?**	
Private transport e.g. own car	670 (87.2)
Public transport bus or train	297 (38.7)
Taxi	125 (16.3)
Plane	135 (17.6)
**Would you fund your own travel if needed?**	
Yes	393 (54.6)
No	25 (3.5)
Maybe	296 (41.1)
Not answered	6 (0.8)
**How much could you afford to pay per month for travel?**	
Nothing	17 (2.4)
Up to £20	114 (15.8)
£21-£50	208 (28.9)
£51-£100	187 (26.0)
Over £100	178 (24.7)
Not answered	16 (2.2)
**Would you be more likely to travel to take part in a clinical trial if all of your travel costs were fully covered?**	
Yes	430 (56.0)
No	64 (8.3)
Maybe	240 (31.3)
Not answered	34 (4.4)
**Has COVID-19 had an impact on your treatment?**	
Yes	186 (24.2)
No	444 (57.8)
Not sure	103 (13.4)
Not answered	35 (4.6)
**Please indicate the impact COVID-19 has had on your treatment:**	
Delayed treatment	91 (11.9)
Lack of access to clinical trials	19 (2.5)
Other	99 (13.0)

### 3.4 Visualisation of patients perspective of trying to enter a clinical trial

Two cartoons drawn by a person living with secondary breast cancer illustrate the patient perspective of trying to enter a clinical trial, and the issues and challenges faced (Supplementary Fig. 1).

### 3.5 Survey free text

Finally, a review of the emergent themes from the survey's free text was carried out to identify quantitative and qualitative data, summarised in Supplementary Table 1.

### 3.6 Qualitative interviews

For details of the qualitative interviews and emerging themes, see Table 5 and supplementary results.

**Table 5:** Thematic content analysis and selected quotes from the 21 qualitative interviews.

Theme 1: The need for information about clinical trials and research
• *‘I think it's hard; you're so busy as a patient, trying to find out information about you and your type of cancer, informing yourself of, you know, what even breast cancer is and all the different acronyms (Interviewee 5).’* • *‘I think it would be really useful if clinical trials were actually spoken about a bit more because apart from me actually asking that question there'd have been nothing at all and I've had a year's treatment since it's been metastatic … but there is literally nothing in the hospital environment. I think that would be really helpful for people because I think some don't even know that clinical trials exist’ (Interviewee 21).* • *‘You're just given information and nobody says, “do you understand … ?” You have to process it yourself. I don't have any nurse to call or to speak to … Not for secondary’ (Interviewee 11).* • *‘I would personally like to have somewhere that you could go for clinical trial information in a layman's language. I don't think even my GP, he doesn't know. It's very difficult to try and find your way through the fog of terminology and whatever to find real, ground-breaking things. Maybe if there was one place that patients could go that for me would be worth researching, like a database, I think [Interviewee 14].’* • *‘I find trawling through the internet trying to find stuff is hard. Yeah, it would be much more helpful to have one place because I'm very interested but sometimes it takes ages … even if patients could be given a web page, or somewhere just to go and have someone to speak to down the line that is up to date with all these trials. Because even the medics don't know all the trials that are going on, do they? Certainly, if their centre isn't involved in it [Interviewee 4].’*

**Theme 2: Barriers to participation**

• *‘Once you get to stage four, is it almost like you're written off, right?’ (Interviewee 17).* • *‘I suppose it feels like metastatic breast cancer is like a little bit written off because we can't survive this' [Interviewee 21].* • *‘Don't use all your medical jargon because it doesn't help us. I got this letter and I had to google every single word; why can't they put it in simple terms? (Interviewee 7)’* • *‘I'm very much up for trials but I'm now at a point where I've outstayed my welcome, I've lived far too long, I've had too many treatment lines and therefore trials, much as though I think it would possibly be of benefit, not necessarily to me but people who will come behind me, I now can't get on one … the longer you live with this disease and the more treatment you have, it precludes you and there are so many other preclusions to clinical trials that part of me thinks, “just how fit do you have to be to get into one?” … I just wonder who this perfect person is at times, you know? Sometimes I don't feel as though it's a very broad spectrum of society (Interviewee 12)’* • *‘I think sometimes for me it's hard to understand why clinical trials need to be so rigid in what their requirements are. Things like, if you don't achieve a biopsy at the time to get on it but you've had the biopsy done only the year before and they're treating you the same. I don't see why they can't then take that biopsy result (Interviewee 18).’* • *‘Secondary breast cancer never gets the attention it needs, even though everybody says it will [Interviewee 7]. ‘*

**Theme 3: Research priorities and hearing good news**

• *‘I think clinical trials are going to become even more important than they ever have been because we are living longer … We need you [health professionals] as much as you need us … why don't you get us to come and give our perspective [Interviewee 12].’* • *‘We're still here, we're not gone, you know … I just think yeah there's a lot more that could be done by us (Interviewee 7). ‘* • *‘The only way things will get better is if we all take part in this, it's kind of a chicken and egg thing, you know? We've got to be part of it and put on the pressure, not just for myself but for other people as well (Interviewee 3).’* • *‘If you look on the internet, [we] are not visible … that's very sad for people because they look on the internet and they think, “well, I'm going to die” … but there are quite a few people out there who are doing very well and they're not visible (Interviewee 2)’* • *‘It's not about sugar coating it or anything like that, it's just about maybe being hopeful, maybe just giving you some good news stories [9]’* • *‘I think it's very positive to hear things about trials. I survive on the knowledge that other people are doing well, you know, success stories. I am not naïve to my diagnosis, I know it's life limiting but I also know that it is treatable … So more of an overview of hope would be something I would really like, more positive stories and people doing well on it would make a lot of difference to a lot of us (Interviewee 13).’*

## 4 Discussion

In this study people living with MBC in the UK were surveyed regarding their experience and views of clinical research. This is one of the largest studies to explore patient's attitudes to clinical research and its relevance heightened by having been co-developed and co-led by a patient living with MBC.

Improving outcomes for patients rest on therapeutic advances delivered via clinical trials [[2], [3], [4], [5], [6], [7]]. However, participation of MBC patients in trials is low, as demonstrated by the small number of respondents reporting trial participation from this study (107; 14 %). This data is consistent with the LIMBER study [24], and a survey of UK adults treated for cancer [25], where 11 %–14 % were offered or had received treatment within a trial.

The vast majority of respondents (86 %) reported knowing what a clinical trial was, indicating a high level of awareness in this cohort. However, only 22 % report a trial being raised by their oncologist, with a higher proportion (33 %) reporting they raised taking part in a clinical trial with their oncologist, indicating a significant number were able to self-advocate for consideration of trials as part of their care. The reasons for the lack of discussion of clinical trials by oncologist was outside of the scope of this study. However, possible reasons include the lack of any relevant trials at the hospital concerned or the lack of trials for their specific clinical circumstances; patient related factors affecting fitness such as performance status or other significant co-morbidities; unwillingness of the clinician to consider a clinical trial or ability to refer externally; pre-judgment that the patient would be unwilling or unable to travel to access a trial. A study to specifically understand the factors in healthcare professionals that influence their decision making to discuss or not clinical trials with patients is vital. Given the pivotal role of healthcare professional such research is key to developing strategies to ensure discussion of clinical trials becomes a standard part of all oncology consultations.

The predominant Caucasian population meant we could not explore differences in experiences or views based on ethnicity. However, the American Black Experience of Clinical Trials and Opportunities for Meaningful Engagement (BECOME) survey which focused primarily on the experience of Black MBC patients in regards to trial participation, and provides some information on differences in the perceptions of trials between black and non-black patients. In this study those self-identifying as black were more likely to believe unstudied treatments may be harmful, less likely to indicate they trust trials as well as trusting that people of all races/ethnicities get fair treatment in trials as compared to non-black participants [26]. The need to specifically to explore the experience of black breast cancer patients in the UK in the context of research is underpinned by data from a secondary data analysis of data from National Cancer Patient Experience Surveys in England [27]. This research found Black African women were less likely to rate their care favourably as compared to White British, Asian.

The key motivating factors reported for participation in a clinical trial within our survey were access to new treatments, helping future patients, playing more active role in own health and more frequent health check-ups. These were similar to the reasons reported by black women, where helping future generations, access to new treatments and closer monitoring were the key reasons to participate in trials [26]. This demonstrates that the core reasons for trial participation are independent of ethnicity. In addition, key reasons reported for not participating in a trial (concerns about side effects and being unsure of potential benefits) closely align with those reported in both black and non-black patients [26] as well as Latina women [28]. While honest information and discussion about the possible risks of entering a trial are key, the provision of information about the mechanism within studies for monitoring and keeping patients safe should also be explained.

The preferred route of information about trials was from a doctor or nurse, with a minority wanting information from a trial database or friends. Black MBC patients were more likely than non-Black patients to want to learn about clinical trials from someone with the same racial or ethnic identity, shared health experience (breast cancer or MBC) or who had been in a clinical trial [26]. Only a minority of non-black patients (11 %) wanted the source to be of the same racial or ethnic identity. These data indicate that the source of trial information may vary for different ethnic groups and that bespoke approaches may be required based on an understanding of that communities’ experience both within society and their experience, and trust, in the healthcare system.

Participants in our survey called for more information about clinical research and particularly highlighted this need during the qualitative interviews. Study participants also requested a database where clinical trial information could be accessed by potential participants, the vast majority indicating they would search a database if it was patient friendly.

A recurrent point made by this population was that they felt “a little bit written off” and their assumed poor survival rates precluded them from the inclusion in clinical trials. There is little evidence about the influence of disease extent on communication about clinical trials and research, for example whether having MBC is more likely to create a barrier. However, this has been suggested by patient advocacy groups [29].

The competitive nature of site selection, as well as the limited number of sites that can be opened, can result in a ‘postcode lottery’ of trial availability at centres, as noted by one participants. Therefore, travelling may be necessary to access a clinical trial. This brings additional costs and financial hardship as was reported by 44 % of cancer patients who participated in clinical trials, most often stemming from travel costs [30]. In our survey only a minority who had participated in a trial reported reimbursement of travel expenses. While a minority of patients were willing to travel to access a clinical trial, this increased to a majority of patients if travel costs were reimbursed. This is consistent with a survey of US adults who reported cost-related considerations would influence decisions to participate in a clinical trial [31]. Addressing and ensuring trial related expenses are covered is an important part of helping to ensure a patient will consider a clinical trial, particularly for patients on lower incomes. Furthermore, comparable survival and toxicity outcomes regardless of geographic proximity from the centre for patients recruited into clinical trials has been reported, with the suggestion that those living further away had lower rates of unplanned hospitalisation [32]. This provides reassurance that distance to the place where a study is conducted does not compromise safety and should not be considered as a barrier. In fact safety may be enhanced as consideration needs to be given to how toxicities might be managed for those living a greater distance from the trial centre.

The women in this group talked about the positive effect of hearing ‘good news’ concerning clinical trials and trial results. Several studies have examined the concept of hope for people with metastatic cancer and other life limiting diseases. More work in this area has been called for but living with a sense of hope and resilience in metastatic cancer has been recognised as a significant factor in assisting individuals to adjust to their experience of living with cancer, reduce psychological distress and enhance wellbeing. There was also a call for much greater awareness in society about MBC. This has been called for by many other patient and patient-interest groups. The MBC Alliance have suggested that the focus on ‘fighting’ and ‘beating’ breast cancer has led to the creation and dominance of a breast cancer ‘survivor’, which ultimately masks the reality that women who have had early-stage breast cancer can develop metastatic disease. Furthermore, this can stigmatise those with MBC [29].

Finally, it is pertinent to point out that the themes these women identified were not mutually exclusive but very closely associated and interdependent. For example, it was considered that better information would enable participant involvement which could, in turn, lift barriers to clinical trial participation and these could both be enhanced by participant involvement [33]. A review of the literature on barriers to inclusion revealed the main barriers were language and communication, lack of trust, access to trials, eligibility criteria, attitudes and beliefs, lack of knowledge around clinical trials, and logistical and practical issues [34]. These themes are evident in our survey results.

Limitations of the current study include that it was based on an electronic survey in English which may have resulted in digital exclusion as well those who were unable to take the questionnaire due to language barriers or literacy issues. The survey participants were predominately white so the results may not reflect some of the challenges experienced by other ethnicities within the UK, particularly those whose first language is not English. In addition, it needs to be noted that the study was carried our whilst some COVD-19 restrictions were still impacting healthcare including trials.

**Fig. 1:**
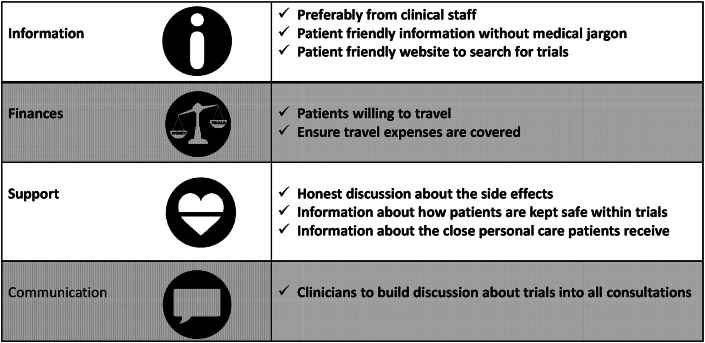
Recommendations to improve participation in clinical trials based on survey results.

In conclusion, clinical research provides an important option for people living with MBC, and our results provide insights into the wishes, experiences and awareness of patients in regard to participation in clinical research. People living with MBC want the opportunity to participate in clinical trials and they highlight the factors that can influence participation. Based on the results of the survey we make a number of recommendations (Fig. 1), that should be taken forward to try and ensure more people living with MBC are not only offered clinical research but also accept such offers. We hope that the results of this survery and the recommendations can inform strategies to improve recruitment into clinical research.

## CRediT authorship contribution statement

**Lesley Stephen:** Writing – review & editing, Writing – original draft, Project administration, Investigation, Conceptualization. **Janet Dunn:** Writing – review & editing, Writing – original draft, Project administration, Methodology, Investigation, Formal analysis. **Claire Balmer:** Writing – review & editing, Project administration, Methodology, Investigation. **Nada Elbeltagi:** Writing – review & editing, Formal analysis. **Sophie Gasson:** Writing – review & editing, Project administration, Methodology, Formal analysis. **Ellen Copson:** Writing – review & editing, Writing – original draft, Project administration, Investigation. **Carlo Palmieri:** Writing – review & editing, Writing – original draft, Supervision, Project administration, Methodology.

## Dedication

This paper is dedicated to the memory of Tassia Haines who worked tirelessly to advocate for and raise the profile of secondary breast cancer. We are grateful to her for the cartoons she drew for this paper to illustrate the patient perspective of clinical trials.

## Availability of data and materials

The datasets generated and analysed during the current study are not publicly available due to participant confidentiality.

## Funding

Funding for this study was provided by a grant from the charity Make Seconds Count and an 10.13039/501100000272NIHR Senior Investigator award (NIHR200274).

## Declaration of competing interests

CP reports grants from Seagen, Daiichi-Sankyo, Pfizer, Exact Science (outside this work); consulting for Seagen, Daiichi-Sankyo, Exact Science, MEDAC, Gilead, Pfizer; honoraria from Pfizer, Seagen, AstraZeneca, Roche, Eisai, Novartis; travel support from Roche, Novartis; leadership role in Advise Make 2nd Count (unpaid). EC reports grants from Astra-Zeneca (outside this work) and research support from SECA; consulting for Pfizer, Novartis; honoraria from Pfizer, AstraZeneca, Roche, Daaichi-Sankyo, Eli-Lilly, Novartis; travel support from Roche, Novartis. LS is a trustee of the charity Make Seconds Count.
