# Supplementary material for: A UK study of the experiences, information needs and attitudes to clinical research among patients living with secondary breast cancer in the UK: A prospective co-developed study
Source: Breast. 2025 Nov 12;85:104644. doi: 10.1016/j.breast.2025.104644 (PMC12670922; doi:10.1016/j.breast.2025.104644)
Supplement: Multimedia component 1 [file mmc1.docx]

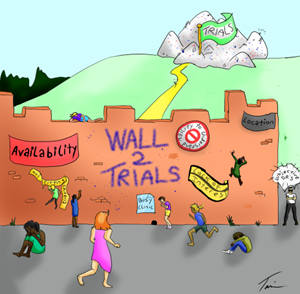


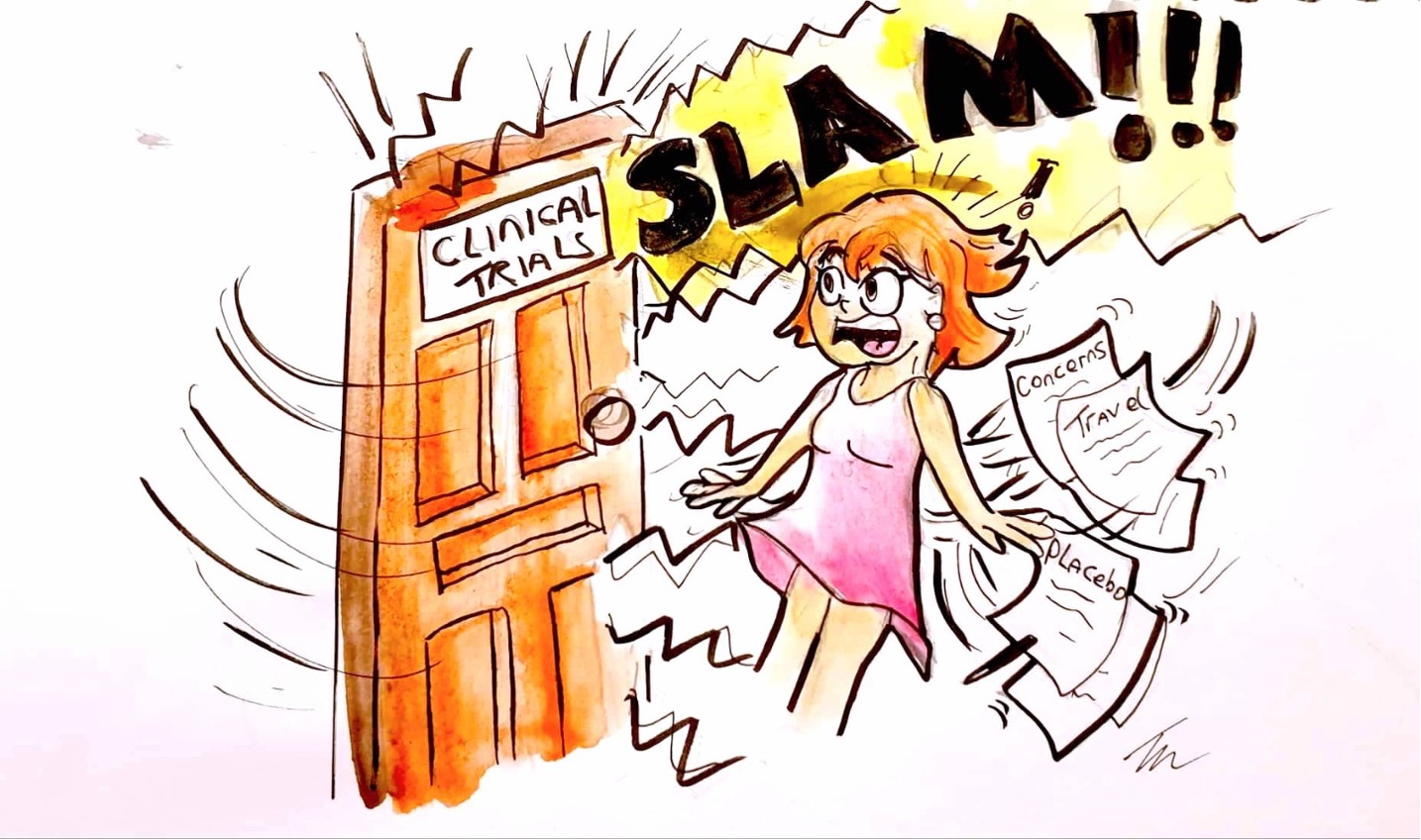


**Supplementary figure 1 Cartoon illustrating from a patient’s perspective the issues and challenges of entering a clinical trial. ©Tessa Haines**
