# Supplementary material for: A UK study of the experiences, information needs and attitudes to clinical research among patients living with secondary breast cancer in the UK: A prospective co-developed study
Source: Breast. 2025 Nov 12;85:104644. doi: 10.1016/j.breast.2025.104644 (PMC12670922; doi:10.1016/j.breast.2025.104644)
Supplement: Multimedia component 2 [file mmc2.docx]

**Supplementary methods**

**Survey questions**

Metastatic Breast Cancer Patients' Experience of Clinical Trials

Start of Block: Default Question Block

Q1


This survey was initiated by a metastatic breast cancer (MBC) patient who has had the opportunity to take part in a clinical trial. Not enough patients are given the opportunity to take part in clinical research and she feels it is important to understand the reasons why.  
 
All answers will be treated confidentially and stored securely in the Warwick University Qualtrics Survey Database.     It will take around 15 minutes to complete and we hope the findings will increase patient awareness of the benefits of clinical research and help improve UK clinical trial practices.     If you would like to take part, please **tick yes** to begin the survey.   Thank you for helping.
 
  


 
 

- Yes
- No

Q2 Please confirm your personal diagnosis

- Diagnosed with primary breast cancer which recurred as secondary (metastatic) breast cancer
- Diagnosed first time with secondary (metastatic) breast cancer

Q3 How long ago were you diagnosed with secondary or metastatic breast cancer?

- Within the last year
- 1 to 2 years ago
- 2 to 3 years ago
- 3 to 4 years ago
- 4 to 5 years ago
- More than 5 years ago

Q4 Please indicate your gender

- Male
- Female
- Prefer not to say

Q5 Please indicate your age group

- Under 30
- 31-40
- 41-50
- 51-60
- 61-70
- 71 or over

Q6 Please specify your ethnicity (tick one):

- Arab
- Asian/Asian British Bangladeshi
- Asian/Asian British Indian
- Asian/Asian British Pakistani
- Any other Asian background
- Black African
- Black Caribbean
- Black - other background
- Mixed - white and Asian
- Mixed - white and black African
- Mixed - white and black Caribbean
- Mixed - other background
- White English/Welsh/Scottish/Northern Irish/British
- White Gypsy or Irish Traveller
- White Irish
- White other background
- Any other ethnic group

Q7 What is your employment status?

- Employed full-time
- Employed part-time
- Self-employed
- Unemployed
- Full-time house wife or husband
- Other (please specify) ________________________________________________

Q8 In order to identify the Regional area where you live, please tell us the first part of your post-code? 
(This will be a mix of letters and numbers eg SW19)

________________________________________________________________

Q9 Which hospital/Cancer Centre are you attending?

________________________________________________________________

Q10 Since being diagnosed with metastatic breast cancer what treatment/s have you had? (tick all that apply)

- Surgery
- Radiotherapy
- Chemotherapy
- Hormone therapy +/- targeted agent eg Abemaciclib, palbociclib, ribociclib or everolimus
- Anti HER2 drugs eg trastuzumab (Herceptin), pertuzumab (Perjeta) or trastuzumab emtansine (Kadcyla)
- Immunotherapy
- Other (please specify) ________________________________________________

Q11 Do you know what a clinical trial is?

- Yes
- No
- Not sure

Q12 Below are some of the potential benefits of taking part in a clinical trial. Please tick any that you were aware of:

- Early access to a potential new cancer treatment
- Playing a more active role in your own health care
- More frequent health check ups as part of your treatment
- Helping future metastatic patients by taking part in today's research

Q13 Has your oncologist ever raised the subject of taking part in a Clinical Trial with you?

- Yes
- No

Q14 Have you ever asked your oncologist about the possibility of taking part in a clinical trial?

- Yes
- No

Q15 What was his/her response?

________________________________________________________________

Q16 How involved do you feel in making decisions about your treatment?

- Very involved
- Slightly involved
- Not at all involved

Q17 What might motivate you to take part in a clinical trial?

________________________________________________________________

Q18 What things might stop you taking part in a Clinical Trial?

- Cost
- Travel
- Unsure of benefits
- Not understanding what the trial is about
- Other - please specify ________________________________________________

Q19  If you were interested in finding out about clinical trials, how would you want to receive that information?

- From a consultant
- From a specialist nurse
- From a friend/another patient
- From a trials database
- No preference

Q20 Have you ever searched a trials registry? (Examples could be the National Cancer Institute registry or clinicaltrials.gov)

- No
- Yes

Q21 Which Cancer Trials Registry did you use?

________________________________________________________________

Q22 Did you find the information you were looking for?

- Yes
- No

Q23 How easy did you find it to use?

|  | Very easy | Very difficult |
| --- | --- | --- |

|  | 0 | 1 | 2 | 3 | 4 | 5 | 6 | 7 | 8 | 9 | 10 |
| --- | --- | --- | --- | --- | --- | --- | --- | --- | --- | --- | --- |

| Slide along this bar to select: | 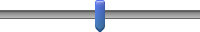 |
| --- | --- |

Q24 How likely would you be to use a patient-friendly secondary breast cancer trials registry?

- Likely
- Unlikely

Q25 Have you ever contacted any of the following organisations for advice on clinical trials? Tick all that apply.

- Make 2nds Count
- Cancer Research UK (CRUK)
- Breast Cancer Now (BCN)
- None
- Other (please specify) ________________________________________________

Q26 Have you ever undergone screening tests (e.g. blood tests or scans) to see if you were eligible to take part in a clinical trial?

- No
- Yes - please specify which tests ________________________________________________

Q27 Have you taken part in a clinical trial?

- No
- Yes

Q28 What was the name of the trial?

________________________________________________________________

Q29 Was your experience of taking part:

- Positive
- Negative
- Unsure

Q30 Please tell us more about your experience of taking part - please highlight things done well or not so well:

________________________________________________________________

Q31 Were your travel expenses reimbursed*?*

- Yes
- No

Q32 Approximately how much were you out of pocket as a result of taking part in a trial?

- More than £500
- £100 - £500
- Up to £100
- I wasn't out of pocket

Q33 Have you ever tried to find out about clinical trials at centres other than your usual hospital or cancer centre?

- Yes
- No

Q34 How did you do this?

________________________________________________________________

Q35 Have you ever asked your oncologist to make enquiries for you at other cancer centres regarding trials for you?

- Yes
- No

Q36 Would you be willing to travel to another cancer centre specifically to take part in a clinical trial?

- Yes
- No
- Maybe

Q37 How far would you be prepared to travel?

- Up to 1 hour
- 1-2 hours
- More than 2 hours

Q38 Would you be prepared to travel abroad?

- No
- Yes but only to a European Country
- Yes to the USA
- Yes world wide

Q39 Within the UK which modes of travel would you be likely to use? Tick all that apply:

- Private transport - e.g. own car
- Public transport - Bus or train
- Taxi
- Plane

Q40 Would you fund your own travel if needed?

- Yes
- No
- Maybe

Q41 How much could you afford to pay per month for travel?

- Nothing
- Up to £20
- £21 - £50
- £51-£100
- Over £100

Q42 Would you be more likely to travel to take part in a clinical trial if all of your travel costs were fully covered?

- Yes
- No
- Maybe

Q43 Has COVID-19 had an impact on your treatment (especially for patients diagnosed in 2020)?

- Yes
- No

Q44 Please indicate the impact COVID-19 has had on your treatment:

- Delayed treatment
- Lack of access to clinical trials
- Other: please describe below ________________________________________________

Q45 Is there anything else you would like to tell us about your experience of COVID-19 and access to clinical trials?

- Yes ________________________________________________
- No

Q46 Is there anything else you would like to tell us about your experience of clinical trials? 


 

________________________________________________________________

Q47 Please can you tell us how you found out about this survey:

- Breast Cancer Now (BCN)
- Cancer Research UK (CRUK)
- Hospital breast care team
- Macmillan
- Maggie's Centre
- Make 2nds Count
- Social Media (add details below) ________________________________________________
- Other please specify below ________________________________________________

Q48       


**Optional:** If you would you be willing to take part in a follow up call to discuss your answers in more detail please provide your name and contact details (email address / phone number) below and one of the research team will get in touch with you to provide further information. Your details will be stored confidentially and only the Research Team dedicated to the survey will have access. 


 
 


 
 

- Name ________________________________________________
- email address ________________________________________________
- Tel number ________________________________________________

Q49
Thank you for taking part in this survey. Your contribution is greatly appreciated.
 
The study has been part-funded by Make 2nds Count, a patient and family focused charity dedicated to giving hope to women and men living with metastatic breast cancer. They fund medical research that will contribute to advancing an increased quality of life for patients.
  
If you want to learn more about how Make 2nds Count can support you, click [here](https://www.make2ndscount.co.uk/)
 
 
 
 


  

End of Block: Default Question Block

**Topic guide**


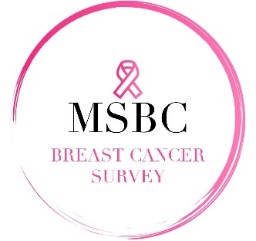


**MSBC Patient telephone Interview Schedule and Topic Guide**

**MSBC: A national study of the experiences, information needs and attitudes to clinical research of patients living with secondary breast cancer in the UK**

**MSBC patient telephone interview– Part of the MSBC study**

Chief Investigator: Janet Dunn

**Section 1: Introduction to the telephone interview study**

**What is the purpose of the telephone interview study?**

The aim of the MSBC telephone interview study is to carry out interviews with study participants to help the research team to understand more fully the experiences, information needs and attitudes to clinical research of patients living with secondary breast cancer in the UK. Through semi-structured interviews we hope to find out more about patients thoughts about research and also how they decided whether or not to take part in trials.

**Method**

We aim to interview around 30 patients. Purposive sampling will be utilised to strive for a mix according to age and socio-demographic characteristics.

Interviews will be conducted by telephone or video conference, whichever is most suitable and convenient for the participant. Interviews will be semi-structured and audio-recorded, using an encrypted device. Recordings will be transferred to a restricted-access drive on the secure University of Warwick network. Some recordings will also be transcribed by University of Warwick researchers. All interviewees will remain anonymous and file names will be pseudonymised. Recordings and transcripts will only be accessible to researchers working on the MSBC study.

**Section 2: Interview Schedule**

**Before switching on the audio-recorder: [Suggested dialogue]**

*‘Firstly, thank you for agreeing to be interviewed as part of the MSBC study. The purpose of the interview is for you to tell us about your* experiences, information needs and attitudes to clinical research*.*

*Before we start talking, I would like to assure you that everything that you say will remain confidential. Your doctor, nurse or other people who work at the hospital will not see or hear any of the information that is shared here. If we do use anything that you have said, such as in a report or journal article, it will be made anonymous so that you cannot be identified.*

*We would like to audio record the interview so we can re-listen and transcribe it. The only people to listen to the recording will be members of the research team at the University of Warwick. The recording will be uploaded to a restricted-access drive on the secure University of Warwick network; once your recording has been transferred to this secure network, it will be erased from the audio-recorder. The computer file of the recording will be deleted at the end of the study. We may transcribe the audio-recording, but your name and anything that could identify you will be removed.*

*Do you have any questions before we switch on the recorder and begin?’*

**Interview body:**

Interviews will be semi-structured to enable interviewees to speak openly and freely about their experiences, their decision whether or not to participate in research as well as their thoughts on clinical trials. Questions, outlined in the topic guide below (Section 3), are there to enable the interviewer to prompt, where necessary, but are not designed to guide or steer the interviewee to give particular responses. They may be used to prompt discussion and to focus on the topic.

**At the end of the interview:**

Thank the interviewee for their help with the study and inform them that a summary of the results will be made available to participants via an end of study report; this will be added to the MSBC website or a copy can be requested from the research team. Study results will also be publicised through breast cancer charities and on the MSBC website.

**Section 3: Interview Topic Guide**

**Interview main body:**

The questions outlined below will be used by the interviewer to prompt discussion, where necessary, and to focus the discussion on the topic. Interviewees will be encouraged to speak freely and openly around these questions:

Schedule for telephone interviews.

Participants will be invited to discuss their experience and the issues that are most important to them regarding involvement in research. However, the interviewer will ask the following (or very similar) questions to ensure that the most important research areas are explored:

| **Question** | **Area of exploration** |
| --- | --- |
| What is your experience of being invited (or not being invited) to take part in a clinical trial and/or research since you have been diagnosed with breast cancer? | Knowledge and experience |
| How did you decide whether or not to take part in the clinical trial and/or research? | Information needs, experience, knowledge, COVID-19 impact |
| Please describe anything you wish you had known before, or since, you were invited to take part, which may have had an impact on your decision? | Barriers, experience, information needs, COVID-19 impact |
| Is there any aspect of your experience with breast cancer that you feel requires, or should be the focus of, future research? | Research priorities and experience |

**Rounding off:**

The interviewer should use the following questions to ensure that the interviewee has had the opportunity to discuss everything they wanted to:

- Is there anything else that you would like to mention?
- Is there anything that you think we should have talked about but haven’t?
- Do you have any questions about the study or about this interview?
