# Supplementary material for: A UK study of the experiences, information needs and attitudes to clinical research among patients living with secondary breast cancer in the UK: A prospective co-developed study
Source: Breast. 2025 Nov 12;85:104644. doi: 10.1016/j.breast.2025.104644 (PMC12670922; doi:10.1016/j.breast.2025.104644)
Supplement: Multimedia component 3 [file mmc3.docx]

**Supplementary Results**

| **Open question** | **Thematically described answer** | **No. of answers attributed to each theme** | **Qualitative description** |
| --- | --- | --- | --- |
| *Have you ever asked your oncologist about the possibility of taking part in a clinical trial?*  32% who answered yes were asked,  **What was his/her response?** | Now is not the time | 52 | “We can look at that option if & when the time comes”  “We will worry about trials when we run out of other options” |
|  | Supportive response | 40 |  |
|  | No trials suitable OR participant unsuitable | 58 |  |
|  | Unsupportive response | 26 | ‘Vague & dismissive’  ‘She said most trials fail & should only be given as a last resort’  ‘He told me to look into trials myself & if I found anything, let him know’ |
| **What things might stop you taking part in a clinical trial?**  (Participants could give more than one reply) | Fear of being given placebo | 18 |  |
|  | Fear of having ineffective/less effective treatment | 8 |  |
|  | Fear of trial drug excluded future treatments | 5 |  |
|  | Fear of having something untried and tested | 3 |  |
| *The 14% who had taken part in clinical trials were asked if their experience had been positive,*  *negative or unsure*  **Please tell us more about your experience of taking part. Please highlight things done well/not so well.** | Positive: Additional / longer monitoring | 10 | ‘I took comfort in regular check ups & scans & having a specialist nurse’  ‘Monitored for longer so mets found sooner’ |
|  | Positive: Additional care (inc psychological) | 9 |  |
|  | Positive: Access to specialist nurse/team | 7 |  |
|  | Negative: Psychological difficulties (e.g. concerning randomisation | 3 | ‘I was concerned about a computer randomly choosing my treatment’ |
|  | Negative: Increased frequency of tests / scans | 3 |  |
|  | Negative: Travel | 3 |  |

**Supplementary table 1 A summary of the emergent themes from the survey’s free text**

**Qualitative interviews**

Twenty-one female participants from all four home nations of the UK were interviewed. Interviews lasted between 9 to 37 minutes. Their ages ranged from 36 to 71 (median 52 years) and their self-reported ethnic backgrounds were one Indian/Asian, one Jewish, one White Danish and eighteen White British. 7 (33%) had been diagnosed with de novo metastatic disease. Information from these interviews was correlated under three final themes, these were (1) The need for information about clinical trials and research; (2) Perceived barriers to clinical trial participation and (3) Participants’ priorities.

**Theme 1: The need for information about clinical trials and research**

The need for more information was reiterated in the qualitative interviews. All participants interviewed called for greater and more accessible information on clinical trials. They expressed dissatisfaction with the information they had been given or accessed. They felt that there was a poor awareness in society generally, as well as little information offered specifically at diagnosis. Health care staff were seen as holding the most trustworthy information by interviewees but they often found it more accessible elsewhere, such as online support groups. When participants were asked how they thought information could be more accessible to them, the overwhelming majority called for a website, database or telephone helpline where relevant and up-to-date information about studies could be held and accessed. Few had access to dedicated MBC nurse specialists and often saw this as a problem to accessing information, see quotes below.

**Quotes:**

I think it's hard; you're so busy as a patient, trying to find out information about you and your type of cancer, informing yourself of, you know, what even breast cancer is and all the different acronyms (Interviewee 5).’

‘I think there needs to be a lot more done for stage four. There seems to be a lot of primary but secondary … ultimately, they know you're going to die’ (Interviewee 4

‘I think it would be really useful if clinical trials were actually spoken about a bit more because apart from me actually asking that question there’d have been nothing at all and I’ve had a year’s treatment since it’s been metastatic … but there is literally nothing in the hospital environment. I think that would be really helpful for people because I think some don’t even know that clinical trials exist’ (Interviewee 21).

‘You're just given information and nobody says, “do you understand…?” You have to process it yourself. I don't have any nurse to call or to speak to ... Not for secondary’ (Interviewee 11).

At worst, this led to participants losing confidence in healthcare staff

‘It made me feel nervous because I wasn’t sure if she was dismissive because there wasn’t anything I could take [but] I also thought, I’ve got friends who are in a similar situation to me who talk about trials a lot and so then I started to question her credibility a bit as if, is she dismissive because she doesn’t know about them? It’s made me worried for further down the line about how actively she will look for treatment that is outside standard protocol. It made me nervous’ (Interviewee 9).

The lack of readily available and trusted information also created misconceptions for participants, with fifteen stating that they believed that clinical trials only available when standard treatment ‘fails’ or 'research is more for people who have run out of options' (Interviewee 7).

‘Without that research and without those clinical trials nothing will change. We will never live long’ (Interviewee 21).

‘I think the medics and the nurses should be talking to people about trials earlier on because I think we all feel that trials are the last resort; right? And I think that is a perception that I was given, that you only go on a trial when everything else fails. That's because it's been never mentioned to me’ (Interviewee 4).

When asked about their experience of being invited to take part in research, many saw it as unnecessary because they hadn’t ‘run out of options’ (Interviewee 7).

‘I haven't been asked to participate in any clinical trials … I went into remission. So, I didn't need any clinical trials because I was okay’ (Interviewee 16).

This was also highlighted by free text survey responses. Of the 32% who said they had asked their consultant about trials, 52 had been told that now was not the time to discuss entry and had been given responses such as, ‘We can look at that option if and when the time comes’ and ‘we will worry about trials when we run out of other options’.

When asked how they thought effective information could be made more accessible to them, the overwhelming majority called for ‘lay’, ‘patient friendly’ and ‘patient-focussed’ information on a database which could hold all the relevant and up-to-date information about studies. Participants called for a website, database or telephone helpline where relevant and up-to-date information about studies could be held and accessed, and that was ‘all in one place'

‘I would personally like to have somewhere that you could go for clinical trial information in a layman’s language. I don't think even my GP, he doesn’t know. It’s very difficult to try and find your way through the fog of terminology and whatever to find real, ground-breaking things. Maybe if there was one place that patients could go that for me would be worth researching, like a database, I think’ (Interviewee 14).

‘I find trawling through the internet trying to find stuff is hard. Yeah, it would be much more helpful to have one place because I’m very interested but sometimes it takes ages … even if patients could be given a web page, or somewhere just to go and have someone to speak to down the line that is up to date with all these trials. Because even the medics don't know all the trials that are going on, do they? Certainly, if their centre isn’t involved in it’ (Interviewee 4).

**Theme 2: Barriers to participation**

A perceived barrier to participation in clinical trials was having metastatic cancer. They felt discounted and that they were given less priority compared to people with primary cancer, due to their assumed limited survival. Occasionally they felt overprotected by 'very well-meaning [but] assumptive' staff. The use of ‘medical jargon’ was seen as a barrier to clinical trial entry and complicated understanding. Accessibility of clinical trials based on geography and ability to travel was also raised. Participants interviewed recognised a ‘postcode lottery’ with some places running more trials and people who lived in certain places facing barriers because of location. Although the survey indicated that many were prepared to travel for treatment, this was described as 'another layer of exclusion’. Another identified barrier was the rigid inclusion and exclusion criteria allowing or preventing potential participants onto clinical trials, see quotes below.

**Quotes**

**Having metastatic breast cancer**

‘Once you get to stage four, is it almost like you're written off, right?’ (Interviewee 17).

‘I suppose it feels like metastatic breast cancer is like a little bit written off because we can’t survive this’ (Interviewee 21).

Participants blamed the media for not giving enough attention, and therefore raising the profile, of metastatic breast Cancer

‘Especially this month, being breast cancer awareness month. There’s just not enough media presence about stage four breast cancer, I’m really concerned about that. It’s like when you get to stage four, you’re written off’ (Interviewee 4]).

**Furthermore, it often led to participants losing confidence in staff**:

‘It’s made me worried for further down the line about how actively she will look for treatment that is outside standard protocol. It’s made me nervous’ (Interviewee 9).

‘Secondary breast cancer never gets the attention it needs, even though everybody says it will’ (Interviewee 7).

Some even felt that clinical staff didn’t give patients with metastatic breast cancer equal attention to those with primary disease either and occasionally, if unintentionally, created barriers themselves to clinical trials access. One woman had seen ‘not for clinical trials’ written on her notes. She felt this was well intentioned as trial entry may have been seen as burdensome for her but she hadn’t been consulted and it was personally unwanted:

Perhaps most worryingly, some participants also felt that clinical staff themselves were influenced by this and may use it as a barrier to clinical trial entry. One participant had seen ‘not for clinical trials’ written on her note.. As a member of staff she felt this was well intentioned and ‘well meaning’ medical staff may have thought being in a trial would have been burdensome for her but she hadn’t been consulted and wondered if this had affected her outcome:

‘I suppose it isn’t until the situation I find myself in now, I kind of think, ‘well, what if?’ I’m not suggesting for a minute it would have made any difference. You know, there are precious few trials for inflammatory breast cancer even on a good year but sometimes you do just think’ (Interviewee 12).

**The use of medical jargon:**

‘Don’t use all your medical jargon because it doesn’t help us. I got this letter and I had to google every single word; why can’t they put it in simple terms?’ (Interviewee 7).

‘If you asked I suspect one hundred people you know, “tell me what a drug trial is?” a lot of them would have no clue … but it’s about the language that is used and we don’t talk about clinical trials until we’re at a point in our lives where it’s really important to understand that’ (Interviewee 12).

**The accessibility of clinical trial based on geography and ability to travel/postcode lottery:**

‘I think there’s a disparity to what people might get in London to what I get up in the North East. So, I think that’s a massive thing. That’s the postcode lottery of what’s available for people’ (Interviewee 8).

‘I’d be happy to be involved in any research but it’s mainly in London’ (Interviewee 7).

What couldn’t be done closer to home?....‘I’m almost loathe to say it but it’s like it’s another layer of exclusion. Not everybody drives, not everybody might have somebody who can take them and you know stay overnight or do something and it just feels as though it’s another exclusion’ (Interviewee 12).

**Rigid inclusion and exclusion criteria allowing or preventing potential participants onto clinical trials:**

‘I’m very much up for trials but I’m now at a point where I’ve outstayed my welcome, I’ve lived far too long, I’ve had too many treatment lines and therefore trials, much as though I think it would possibly be of benefit, not necessarily to me but people who will come behind me, I now can’t get on one … the longer you live with this disease and the more treatment you have, it precludes you and there are so many other preclusions to clinical trials that part of me thinks, “just how fit do you have to be to get into one?” … I just wonder who this perfect person is at times, you know? Sometimes I don’t feel as though it’s a very broad spectrum of society’ (Interviewee 12).

‘I think sometimes for me it’s hard to understand why clinical trials need to be so rigid in what their requirements are. Things like, if you don't achieve a biopsy at the time to get on it but you've had the biopsy done only the year before and they're treating you the same. I don't see why they can't then take that biopsy result’ (Interviewee 18).

**Theme 3: Research priorities**

Participants interviewed were asked what their own priorities for research were. Many talked about personal interests and priorities (e.g. complementary treatments, genetic links) or advocated for a database of relevant clinical trial information. However, there were two further important priorities for these participants. One was participant involvement and a feeling of responsibility to be involved to inform others, including medical staff, and another was a call for more positivity and ‘good news stories’ concerning research for those with metastatic disease. They were encouraged by positivity but felt having a diagnosis of MBC was often linked to hopelessness and negativity, quotes below.

**Quotes**

‘Other doctors need to be updated as well. Not making excuses for them but patients need to say, there is that trial, I'll talk to my doctor about it’ (Interviewee 11).

‘Why don’t you get us advocates on stage and we can have a clinical dialogue with the people that matter?’

‘I think clinical trials are going to become even more important than they ever have been because we are living longer … We need you [health professionals] as much as you need us and why don’t you get some us to come and give our perspective’ (Interviewee 12).

‘We're still here, we're not gone, you know … I just think yeah there's a lot more that could be done by us’ (Interviewee 7).

‘The only way things will get better is if we all take part in this, it’s kind of a chicken and egg thing, you know? We’ve got to be part of it and put on the pressure, not just for myself but for other people as well’ (Interviewee 3).

**Wanting to hear good news**

‘If you look on the internet, [we] are not visible … that’s very sad for people because they look on the internet and they think, “well, I’m going to die” … but there are quite a few people out there who are doing very well and they’re not visible’ (Interviewee 2).

‘It's not about sugar coating it or anything like that, it’s just about maybe being hopeful, maybe just giving you some good news stories’ (Interviewee 9.)

‘I think it’s very positive to hear things about trials. I survive on the knowledge that other people are doing well, you know, success stories. I am not naïve to my diagnosis, I know it’s life limiting but I also know that it is treatable … So more of an overview of hope would be something I would really like, more positive stories and people doing well on it would make a lot of difference to a lot of us’ (Interviewee 13).
